# Interstitial Chronic Hepatitis

**Published:** 1882-10

**Authors:** 


blossoms of this part will close; afterward the anæsthetic effect will be observed on the middle part of the plant, until at last the whole plant is anæsthetized.—*Lyon Médicale.*

INTERSTITIAL CHRONIC HEPATITIS.

The results of careful investigations which Dr. Maffuci made on this disease are as follows: 1. Atrophic hepatic cirrhosis is always caused by periphlebitis of the vena porta. 2. This periphlebitis is reproduced either by chemical agents, or by septic matter circulating in the blood of the vein, or by mechanical irritants which operate on the tunica adventitia. 3. The inflammatory process is similar to that in other organs; it is characterized by hyperæmia with migration of leucocytes, and by formation of exsudates, in clinical as well as in experimental observations. 4. The periphlebitis is entirely different from the interstitial inflammations of the liver provoked on the biliary ducts. 5. The profound troubles of nutrition of the hepatic arteries cause necrobiosis of the liver. 6. The slighter nutritive alterations of these vessels can effect only an active hyperæmia. 7. The interstitial alterations of the liver provoked by arterial lesions are very different from those produced by periphlebitis. 8. These alterations must be taken into consideration in most observations.—*H. Movimento and Lyon Médicale.*

